# Predictors of Inappropriate Proton Pump Inhibitors Use in Elderly Patients

**DOI:** 10.1155/2019/7591045

**Published:** 2019-01-01

**Authors:** Panagiota Voukelatou, Ioannis Vrettos, Georgia Emmanouilidou, Konstantinos Dodos, Georgia Skotsimara, Dimitra Kontogeorgou, Andreas Kalliakmanis

**Affiliations:** 2nd Department of Internal Medicine, General and Oncology Hospital of Kifissia “Agioi Anargyroi”, Athens, Greece

## Abstract

*Introduction. *Overutilization of Proton Pump Inhibitors (PPIs) both in ambulatory care and in the inpatient setting possesses economic implications and increases the risk for adverse drug reactions. This study was undertaken to identify factors associated with inappropriate PPI use among consecutively unplanned admissions of elderly patients at the time of admission.* Materials and Methods.* In 758 patients (54.2% women), mean age 80.3±8.0 (M±1SD), demographic characteristics, and medical and medication history were recorded. Parametric tests and multiple logistic regression analysis were applied to identify the predictors of inappropriate PPI use.* Results.* 232 patients (30.6%) were receiving PPIs. 37 (4.9%) were receiving PPIs appropriately and 195 (25.7%) were receiving PPIs without a proper indication. Consequently, PPIs prescribing was inappropriate in 195/232 (84%). Moreover, 512 patients (67.5%) were not receiving PPIs appropriately and 14 patients (1.8%) were not receiving PPIs but they had a proper indication. When we compared patients receiving PPIs without a proper indication with those who were not receiving PPIs, a statistical difference was found according to Charlson Comorbidity Index (p≤0.001, U=37922.00), number of diseases (p≤0.001, U=33269.00) and medications (p≤0.001, U=31218.50), Katz Index score (p=0.01, U=45328.00), and the use of blood thinners (p≤0.001, *χ*^2^=21.15). In multivariate analysis the only independent predictor of inappropriate PPI use was the number of medications (p=0.001, OR=1.16, 95%CI 1.06-1.27).* Conclusions.* The main predictor of inappropriate PPI use was the number of received medications. Εfforts needed to apply the predefined criteria for PPI prescription and to deprescribe PPIs received inappropriately.

## 1. Introduction

Concern about overutilization of PPIs both in ambulatory care and in the inpatient setting has been growing [1]. This phenomenon possesses economic implications [2] and also increases the risk for adverse drug reactions [3]. The overall benefits of therapy with a PPI significantly outweigh potential risks in most patients. However, when they are prescribed without a clear clinical indication they only expose patients to the risks of prescription [4]. This is especially important for the elderly who are prone to the phenomenon of polypharmacy [5]. In this population when a PPI without indication is added to the medication list, the risk for both adverse drug events [6] and drug to drug interactions [7] increases.

In general, data concerning the factors associated with inappropriate PPI use in ambulatory setting, especially in the elderly, are scarce. The present study was undertaken to identify factors associated with inappropriate PPI use among consecutively unplanned admissions of elderly patients, in a department of internal medicine.

## 2. Materials and Methods

### 2.1. Study Population

A prospective case-control study was carried out among patients older than 65 years, consecutively admitted to the internal medicine ward through the emergency department of General and Oncological Hospital of Kifissia “Agioi Anargyroi” during March 2015 to April 2018. Patients' demographic characteristics (age, gender), medical history (comorbidities, physical activity status), and medication history (number and names of medications) were recorded by a study physician at admission. If a patient was receiving treatment with a PPI, we tried to identify the indication and moreover to ascertain if the indication was in accordance with NICE guidelines [8]. According to that, patients were categorized into four groups: those who were receiving PPIs appropriately, those who were receiving PPIs without a proper indication, those who were not receiving PPIs appropriately, and those who were not receiving PPIs but had a proper indication. Comorbidity was measured using the Charlson Comorbidity Index (CCI) [9] and physical activity status was assessed using the Katz Index [10]. Information regarding demographic characteristics, medication history, and medical history was obtained by interviewing either the patients or their relatives, when patients were not able to communicate. Verification was made through the state electronic databases that provide information about the prescribed medications. The study was approved by the institutional ethical and scientific committee. An informed consent was obtained from each patient who agreed to participate or from their relative.

### 2.2. Definition of Inappropriate PPI Use

According to NICE guidelines PPIs are indicated for patients with severe gastro-oesophageal reflux disease or for those with proven pathology (oesophageal ulceration, Barrett's oesophagus), for patients with documented duodenal or gastric ulcers (*H. pylori*, nonsteroidal anti-inflammatory drug, or aspirin induced ulcers and ulcers due to rare causes such as Zollinger–Ellison syndrome or Crohn's disease), and for patients with uninvestigated dyspepsia, with nonulcer dyspepsia, or with mild symptoms of dyspepsia for a short, low dose course to assess response. Any other indication for PPI use is considered inappropriate [8].

### 2.3. Statistical Analysis

For categorical variables, frequencies and percentages were reported. The Shapiro-Wilk test was used to assess the normality of distribution of continuous variables. We found that age was normally distributed while CCI, Katz index, number of diseases, and number of medications had a non-Gaussian distribution. Age is expressed as means ± 1 standard deviation (M±1SD) while the rest of the continuous variables are expressed as means ± 95% confidence interval (95% CI). For categorical variables, a chi-square test was employed to evaluate whether inappropriate PPI use differed across patients' demographic characteristics and across patients' medical and medication history. Age was analyzed both as a continuous and as a categorical variable and categorized into three groups (65-74, 75-84, and ≥85 years old). Student's t-test was used to compare age and Mann-Whitney U test was used to compare CCI, Katz index, number of diseases, and number of medications between patients receiving PPIs without a proper indication and those who were not receiving PPIs. Those who were receiving PPIs appropriately were excluded from the analysis. Furthermore, for the statistical analysis, in patients receiving PPIs without indication the number of medications did not include the PPI. A value of p<0.05 was considered significant. Variables that had a significant influence on inappropriate PPI use in the bivariate analysis were included in the separate logistic regression analysis to identify the most important ones. Concerning the diagnostics of the model there is a significant reduction in residual deviance. At 754 degrees of freedom the value measures 920.90 whereas at 749 it measures 766.13. The difference in residual deviance between the null and the full model is 163.87 with a p value (of *χ*^2^ test) <0.01 smaller than 0.05. IVFs appear to be smaller than 4, so multicollinearity is not present. Model fit was checked via Hosmer and Lemeshow test with a p value smaller than 0.001, fact that confirms the goodness of fit in our model. Furthermore backward elimination was conducted and yielded an ACI score of 778.13 with 749 degrees of freedom. By the gradual elimination of the variables the AIC becomes higher, so we can conclude that the model we used is the best one that can be implemented in this database. By means of logistic regression prediction model, the most important predictors of inappropriate PPI use are presented as odds ratios (OR), including 95%CI. All analysis was performed using SPSS v22.0.

## 3. Results

During the study period, 758 patients older than 65 years were admitted to the medical unit through the emergency department. Response rate was 100% since all patients agreed to participate. Mean age was 80.3±8.0 (M±1SD, range 65–103). There were 411 women (54.2%) and 347 men (45.8%). 232 (30.6%) were receiving PPIs. From those 37 patients (4.9%) were receiving PPIs appropriately and 195 patients (25.7%) were receiving PPIs without a proper indication. Consequently, PPIs prescribing was not concordant with the recommendations in 195 out of 232 patients (84%). Moreover, 512 patients (67.5%) were not receiving PPIs appropriately and 14 patients (1.8%) were not receiving PPIs despite the fact that they had a proper indication. The demographic and medical history differences between patients receiving PPIs without a proper indication and those that were not receiving PPIs are presented in Table 1.

Statistical difference was found when we compared patients receiving PPIs without a proper indication to those who were not receiving PPIs, according to CCI, number of diseases and received medications, Katz index score, and the use of blood thinners. In multivariate analysis the only independent predictor of inappropriate PPI use was the number of received medications (p=0.001, OR=1.16, 95%CI 1.06-1.27). The full model results are presented in Table 2.

**Figure 1:**
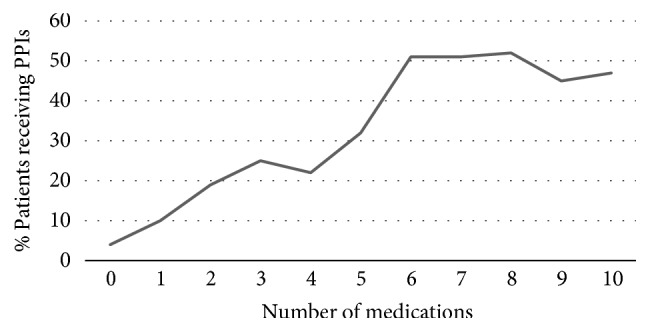
Percentage growth of patients receiving PPIs over the number of medications.

As shown in Figure 1, the percentage of patients receiving PPIs increases proportionally with the number of medications. More specifically the percentage of patients receiving PPIs increases till 5 medications, while over 6 the percentage remains almost stable, around 50%.

## 4. Discussion

According to the results of the present study, among elderly patients requiring hospitalization up to one out of four received PPIs without a clear indication. Among those who were receiving PPIs only 16% were in concordance with the recommendations. In those patients inappropriate PPI use was mainly linked to the number of medications received.

In previous studies inappropriate prescribing of PPIs was observed in 54-70% among patients receiving PPIs at the time of hospital admission [11–15]. This proportion was approximately 60% in elderly patients requiring hospitalization [14, 15]. The proportion of patients receiving PPIs inappropriately was greater in our study. This is not surprising since, in a previous study from Greece concerning patients discharged from the hospital, PPIs were prescribed for a licensed indication in 18.8% of the patients, less than that reported in studies from other countries [16].

In studies addressing factors related to non-guideline recommended prescribing of PPIs several factors have been reported. Those factors can be categorized in medication related (anticoagulant therapy [17], antidepressants [18], and anticholinergic drugs [19]), disease related (dementia [15], osteoarthritis [18], and osteoporosis [18]), and patient related (number of chronic diseases [18], number of medications [14, 19, 20], female sex [20], nursing home residence [20], and lower CCI [15]). As it is highlighted in our study, three previous studies addressed the fact that the number of medications is strongly related to inappropriate PPI use [14, 19, 20] and two of these referred to elderly population [14, 19]. This is not surprising since it has already been reported that, for each additional drug in the medication list, the chance of long-term PPI use increases by almost 30% [20]. Besides, Moriarty et al. has already concluded that long-term maximal-dose PPI prescribing in older adults is not consistently associated with gastrointestinal bleeding risk [21].

Our study has some limitations. At first the study design did not allow the generalization of conclusions concerning the prevalence of inappropriate PPI prescribing in the elderly in other settings, since the sample originates from the hospital. Another limitation of this study was the fact that it was carried out in a single internal medicine ward of a tertiary care hospital. Nevertheless, we believe that the patients profile did not vary from that of patients admitted through the emergency departments to the internal medicine wards of other tertiary hospitals and consequently the sample is representative for this patients' population. A third limitation was the fact that we did not analyze separately all medical conditions of the patients in order to identify whether some of these were significantly associated with inappropriate PPI use. This was performed considering the fact that CCI is more preferable than analyzing every medical condition separately, as it is an index of diseases' burden. Finally the appropriate dosing was not considered, so the prevalence of inappropriate prescribing could have been even higher.

## 5. Conclusions

Our study points out that the main predictor of inappropriate PPI use in the elderly was the number of received medications. In other words, it is more possible for a physician to prescribe a PPI when his patient receives too many drugs. In this occasion patients are exposed to the risks of prescription without any expected benefit. Εfforts must be made to apply the predefined criteria for PPI prescription and to deprescribe PPIs received inappropriately.

## Figures and Tables

**Table 1:** Comparison of demographic and medical history differences between patients receiving PPIs without a proper indication and patients who were not receiving PPIs.

**Demographic characteristics & Medical history**	Patients receiving PPIs without a proper indication n=195 (25.7%)	Patients not receiving PPIs n=526 (74.3%)	Statistical significance
**Gender **			
Males	94 (48.2%)	238 (45.2%)	NS
Females	101 (51.8%)	288 (54.8%)	

**Age **(M±1SD) (years old)	80.0±7.9	80.4±8.0	NS

**Age group**			
65-74 (years old)	51 (26.1%)	131 (24.9%)	NS
75-84 (years old)	83 (42.6%)	209 (39.7%)	
≥85 (years old)	61 (31.3%)	186 (35.4%)	

**Katz index (95**%** CI)**	3.36 (3.01-3.70)	3.88 (3.65-4.09)	p=0.01 (U=45328.00)

**CCI (95**%** CI)**	6.18 (5.90-6.46)	5.36 (5.18-5.54)	p ≤0.001 (U=37922.00)

**Number of diseases (95**%** CI)**	3.77 (3.56-3.99)	2.73 (2.59-2.86)	p ≤0.001 (U=33269.00)

**Number of medications (95**%** CI)**	6.03 (5.64-6.43)	4.10 (3.86-4.35)	p ≤0.001 (U=31218.50)

**Blood thinner **			
*Yes*	111 (56.9%)	199 (37.8%)	p ≤0.001
*No*	84 (43.1%)	327 (62.2%)	(*χ*^2^=21.15)

NS: nonsignificant; CI: confidence interval; CCI: Charlson Comorbidity Index; mean ± 1 standard deviation.

**Table 2:** Multivariable binary logistic regression prediction model.

**Variables **	**p-value**	** OR**	** 95**%** CI**	
			**Lower **	**Upper**
Number of diseases	0.09	1.14	0.98	1.34

Number of medications	0.001	1.16	1.06	1.27

Katz index	0.10	0.94	0.88	1.01

CCI	0.08	1.08	0.99	1.19

Blood thinner	0.17	1.30	0.89	1.90

CI: confidence interval; CCI: Charlson Comorbidity Index; OR: odds ratio.

## Data Availability

The data used to support the findings of this study are included within the article.
